# Microglial neuroinflammation contributes to tau accumulation in chronic traumatic encephalopathy

**DOI:** 10.1186/s40478-016-0382-8

**Published:** 2016-10-28

**Authors:** Jonathan D. Cherry, Yorghos Tripodis, Victor E. Alvarez, Bertrand Huber, Patrick T. Kiernan, Daniel H. Daneshvar, Jesse Mez, Philip H. Montenigro, Todd M. Solomon, Michael L. Alosco, Robert A. Stern, Ann C. McKee, Thor D. Stein

**Affiliations:** 1 Boston University Alzheimer’s Disease and CTE Center, Boston University School of Medicine, Boston, MA USA; 2 Department of Neurology, Boston University School of Medicine, Boston, MA USA; 3 Department of Biostatistics, Boston University School of Public Health, Boston, MA USA; 4 Department of Anatomy and Neurobiology, Boston University School of Medicine, Boston, MA USA; 5 Department of Neurosurgery, Boston University School of Medicine, Boston, MA USA; 6 Department of Pathology and Laboratory Medicine, Boston University School of Medicine, Boston, MA USA; 7 VA Boston Healthcare System, Boston, MA USA; 8 Department of Veterans Affairs Medical Center, Bedford, MA USA

**Keywords:** CTE, Neuroinflammation, Microglia, Repetitive head impacts, Mild traumatic brain injury, American football

## Abstract

The chronic effects of repetitive head impacts (RHI) on the development of neuroinflammation and its relationship to chronic traumatic encephalopathy (CTE) are unknown. Here we set out to determine the relationship between RHI exposure, neuroinflammation, and the development of hyperphosphorylated tau (ptau) pathology and dementia risk in CTE. We studied a cohort of 66 deceased American football athletes from the Boston University-Veteran’s Affairs-Concussion Legacy Foundation Brain Bank as well as 16 non-athlete controls. Subjects with a neurodegenerative disease other than CTE were excluded. Counts of total and activated microglia, astrocytes, and ptau pathology were performed in the dorsolateral frontal cortex (DLF). Binary logistic and simultaneous equation regression models were used to test associations between RHI exposure, microglia, ptau pathology, and dementia. Duration of RHI exposure and the development and severity of CTE were associated with reactive microglial morphology and increased numbers of CD68 immunoreactive microglia in the DLF. A simultaneous equation regression model demonstrated that RHI exposure had a significant direct effect on CD68 cell density (*p* < 0.0001) and ptau pathology (*p* < 0.0001) independent of age at death. The effect of RHI on ptau pathology was partially mediated through increased CD68 positive cell density. A binary logistic regression demonstrated that a diagnosis of dementia was significantly predicted by CD68 cell density (OR = 1.010, *p* = 0.011) independent of age (OR = 1.055, *p* = 0.007), but this effect disappeared when ptau pathology was included in the model. In conclusion, RHI is associated with chronic activation of microglia, which may partially mediate the effect of RHI on the development of ptau pathology and dementia in CTE. Inflammatory molecules may be important diagnostic or predictive biomarkers as well as promising therapeutic targets in CTE.

## Introduction

Chronic traumatic encephalopathy (CTE) is a progressive neurodegenerative disease associated with a prolonged history of repetitive head impacts (RHI) including concussive and subconcussive hits [1, 2]. Athletes participating in sports such as American football, hockey, boxing, soccer, and rugby, as well as military personnel, may be at risk due to years of exposure to head impacts [1, 2]. The clinical features of CTE typically manifest years or decades after exposure to RHI and consist of impairments in mood, behavior, cognition, and motor functioning [3]. Recently, consensus criteria have established CTE as a distinct tauopathy defined by abnormally phosphorylated tau (ptau) accumulation within neurons, astrocytes, and cell processes in an irregular and patchy distribution that is perivascular and concentrated within the depths of sulci [4]. The earliest pathological changes of CTE are often observed within the sulcal depths of the dorsolateral frontal cortex. In later, more severe stages, ptau pathology is present within neighboring cortical regions and within medial temporal lobe structures such as the hippocampus [4, 5].

Growing evidence suggests that cumulative RHI exposure is associated with the development and increased severity of CTE [3, 5–8]. We have previously shown in a heterogeneous cohort of deceased contact sport athletes and military personnel that the number of years of exposure to RHI significantly predicts increased CTE stage as defined by the extent of ptau pathology [5, 9]. Furthermore, a cumulative head impact index based on position, level of play, and impact frequencies from helmet accelerometer studies in American football players was associated with later-life cognitive and neurobehavioral impairment [8]. In contrast, the reported number of concussions was not significantly correlated with CTE stage [5, 9] and was less predictive than the cumulative head impact index of cognitive and neurobehavioral impairment [8]. Furthermore, 16 % of individuals diagnosed with CTE had no reported history of concussions suggesting subconcussive hits are sufficient for the development of the disease [10]. However, to date, an association between years of contact sports play and a quantitative measure of ptau pathology in the brain has not been shown, and the underlying factors linking RHI exposure to the development of CTE are unknown.

Although mild traumatic brain injury has been associated with multifocal axonal loss and microglial activation [1], the factors that initiate the development of ptau pathology are unknown. While the neuroinflammatory response to mild acute insults is usually short-lived [11, 12], RHI may lead to chronic neuroinflammation that induces a self-perpetuating inflammatory cycle with longstanding activation of microglia, including sustained release of inflammatory mediators [13]. Recent evidence suggests that neuroinflammatory cytokines and reactive microglia exacerbate tau pathology and contribute to the spreading of ptau in rodent models of Alzheimer disease and other tauopathies, suggesting a potential link between traumatic brain injury and CTE [14, 15]. However, the neuroinflammatory state years after a period of RHI is unknown.

Here, we test the hypothesis that increased neuroinflammation, defined by an increased astrocyte and microglial number and an upregulation of the inflammatory/phagocytic marker CD68, is associated with a history of longer exposure to RHI, increased CTE ptau pathology, and increased risk of developing dementia in a cohort of American football players and non-exposed control subjects.

## Materials and methods

### Subjects

A total of 66 brains from former American football players with a history of RHI from the VA-BU-CLF Brain Bank were neuropathologically evaluated for the changes of CTE, as well as other neurodegenerative conditions using previously published selection criteria and protocols [16]. Subjects were selected from the entire 297 subjects who had donated to the brain bank based on the following criteria: 124 subjects were excluded due to carrying a neuropathological diagnosis of either Alzheimer’s disease, Parkinson’s disease, Dementia with Lewy bodies, frontotemporal lobar degeneration, or motor neuron disease; an additional 65 subjects lacked any history of playing American football and were removed; 10 subjects were excluded for no having documentation of the length of contact sport exposure; 30 subjects did not have sufficient DLF tissue for analysis; 2 subjects were excluded due to a gunshot wound through the tissue preventing proper analysis. All cases selected were male. Sixteen additional brains from non-athlete controls were obtained from the Framingham Heart Study. Subjects were selected as controls based on their lack of history of contact sport play and lack of neurodegenerative disease other than CTE (however, no subjects without a history of contact sports play had CTE). Clinical assessment details are provided below. Next of kin provided written consent for participation and brain donation. IRB approval for brain donation was obtained through the Boston University Alzheimer's Disease Center (BU ADC) and CTE Center and Edith Nourse Rogers Memorial Veterans Hospital, Bedford, MA. Of the 66 former American football players, 18 players were found to have no neurodegenerative pathology after comprehensive neuropathological examination and were designated “RHI with no CTE” and 48 players were diagnosed with CTE using recently published NINDS criteria for neuropathologic diagnosis of CTE [5]. Of the subjects with CTE, 13 subjects were diagnosed with stage I or II CTE (Mild CTE) using the McKee staging criteria [5] and 35 subjects were diagnosed with stage III or IV CTE (Severe CTE). Table 1 contains exposure characteristics for each group.

**Table 1:** Demographic and exposure characteristics of subject groups

	*n*	Mean age at death (yrs)	Age of first exposure to RHI (yrs)	Years exposure to head injury	Number of reported concussions	Age of symptom onset	Cases with dementia
Controls	16	76 ± 3	N/A	0	0.1 ± 0.1	N/A	0.00 %
RHI without CTE	18	32 ± 4	10 ± 0.5	9 ± 1	30.1 ± 9.2	27 ± 4	5.56 %
CTE Mild (Stage I & II)	13	44 ± 6	14 ± 1	15 ± 0.8	41.5 ± 16.4	34 ± 6	7.69 %
CTE Severe (Stage III & IV)	35	66 ± 2	12 ± 0.5	17 ± 0.7	38.4 ± 12.6	48 ± 3	48.57 %

Data expressed as mean ± SEM

### Clinical assessment

Institutional Review Board approval for post-mortem clinical record review, interviews with family members, and neuropathological evaluation was obtained through Boston University School of Medicine. RHI history, concussive events, athletic or military service history, history of cognitive, mood, and behavior changes, and clinical status leading up to death were assessed with post-mortem interviews with informants and through online surveys and medical record review as described previously [16]. RHI exposure was defined by the total numbers of years football was played. All interviews were performed by neurologists and neuropsychologists trained to assess for RHI exposure and neurodegenerative diseases. To obtain additional clinical information, medical record review was performed. Through the above methods, age of cognitive, behavior, and mood symptom onset were ascertained [16]. Presence of dementia was determined based on postmortem interviews with next of kin asking if a physician gave a diagnosis of dementia during life. All interviews were conducted independently and blinded to the results of the neuropathological examination. An identical assessment was performed for the Framingham Heart Study cohort.

### Neuropathological examination

Neuropathological processing followed the procedures previously established for the BU ADC and CTE brain bank, which included a comprehensive analysis designed to screen for neurodegenerative conditions [16, 17]. The neuropathological diagnosis of CTE was made using the NINDS consensus criteria for CTE [4] that are based on the presence of abnormal perivascular accumulations of hyperphosphorylated tau in neurons, astrocytes, and cell processes in an irregular and patchy distribution concentrated at the depths of cortical sulci [4]. The McKee stages of CTE, varying from stage I to stage IV, are based on the extent and severity of the ptau pathology [5]. Cases that met neuropathological criteria for comorbid Alzheimer disease, frontotemporal lobar degeneration, diffuse Lewy body disease, Parkinson’s disease, or motor neuron disease were excluded from analysis.

### Immunohistochemistry

All brain tissue was processed identically by fixation in periodate-lysine-paraformaldehyde and stored at 4 °C. A tissue block from the dorsolateral frontal cortex was taken perpendicular to the superior frontal sulcus, embedded in paraffin and cut at 10 μm (AT8) or 20 μm (Iba1, CD68, GFAP). Antigen retrieval was performed by boiling sections in citrate buffer (pH 6.0) for 10 mins. Sections were incubated at 4 °C overnight with antibodies to anti-Iba1 (Wako, 1:500), anti-GFAP (Dako, 1:500), anti-PHF-tau (AT8) (Pierce Endogen, 1:2000), and anti-CD68 (Vector, 1:500). Sections were treated with biotinylated secondary antibodies then labeled with a 3-amino-9-ethylcarbazol HRP substrate kit (Vector Laboratories). Sections were counter stained with Gill’s Hematoxlin (Vector Laborities H-3401) and coverslipped with Permount mounting medium. For fluorescent stains, antibody visualization was performed using secondary antibodies bound to Alexa fluorophores (Invitrogen) at a dilution of 1:500. Sudan black at a dilution of 0.1 % was used to quench autofluorescence.

### Microscopy and analysis

The sulcal depths of the dorsolateral frontal (DLF) cortex are often the first regions involved by CTE pathology [5], suggesting this region could be a sensitive measure of early changes. Immunostained slides were scanned and digitized at 20× magnification using the Aperio ScanScope (Leica) as previously described [18]. Serial sections were cut and used for the markers mentioned above. Each stain and quantification was performed on one section using subsequent serial sections in the order of AT8, Iba1, GFAP, and then CD68. The depth of the cortical sulcus (defined as the bottom third of two connecting gyri) was selected and highlighted in ImageScope (Lecia). The white matter/gray matter boundary was used as the outer edge of the region of interest so only gray matter was highlighted. Using Leica image analysis and automated counting software, the Aperio nuclear algorithm (Version 9) was set to recognize and count Iba1+ microglia, GFAP+ astrocytes, CD68 positive cells, and AT8 immunoreactive neurofibrillary tangles (NFTs) restricted to the highlighted areas. Each counting algorithm was individually modified to recognize cell shape, size, and staining intensity. The Aperio positive pixel count (Version 9) was also used to determine the area of immunoreactivity. All quantifications were standardized to the area measured and presented as density per analyzed area.

### Statistics

Statistical analysis was performed with SPSS version 20.0 (IBM inc.), Prism v6 (Graphpad Software), and SAS version 9.4 (SAS Institute). CD68 density, AT8 immunoreactive NFT density, and AT8 density were log transformed to normalize for regression analyses. A one-way ANOVA was used to compare Iba1 and GFAP cell density among control, RHI, and CTE groups. CD68 density was compared using a Kruskal-Wallis test due to the non-normal distribution of the data. Age at death was included in all regression analyses to control for age-associated changes. Since the outcome variables (CD68 cell density and AT8 density) are correlated, the use of independent regression models for each outcome would give biased estimates. To avoid this endogeneity problem, simultaneous equation modeling was used to determine direct and indirect effects of predictor variables (age at death and years of RHI exposure) on outcome variables (CD68 cell density and AT8 density). Since we have two exogenous variables (age at death and years of RHI exposure) for two outcomes (CD68 cell density and AT8 density), the model is just-identified. Years of RHI was used as a metric of exposure as opposed to number of concussion due to the previous finding CTE tauopathy stage was significantly predicted by years of contact sports play, but not number of concussions [10]. To ascertain correlations between dementia and neuroinflammation, we elected to use age of first exposure to football as the beginning of the pathologic process, as CTE pathology likely exists on a continuum. AT8 immunoreactive NFT density and CD68 cell density were plotted based on time from first exposure to death and a linear regression was used to test for correlations (Fig. 3). A probability curve was generated using a binary logistic regression predicting dementia based on time from first exposure to death. Another binary logistic regression was then used to test the association between dementia, CD68 cell density, and AT8 immunoreactive NFT density.

## Results

### Subjects without CTE had less RHI exposure

One-way ANOVA analysis demonstrated individuals with RHI exposure but no CTE pathology were significantly younger at age of death compared to those with severe CTE (*p* < 0.001) but not mild CTE (*p* = 0.120). Furthermore, the RHI without CTE group had significantly less years of exposure to RHI than both mild (*p* = 0.002) and severe (*p* > 0.001) CTE. No significant difference was observed for number of reported concussions between the RHI exposed groups (Table 1).

### Increased neuroinflammation is associated with longer RHI exposure

**Fig. 1:**
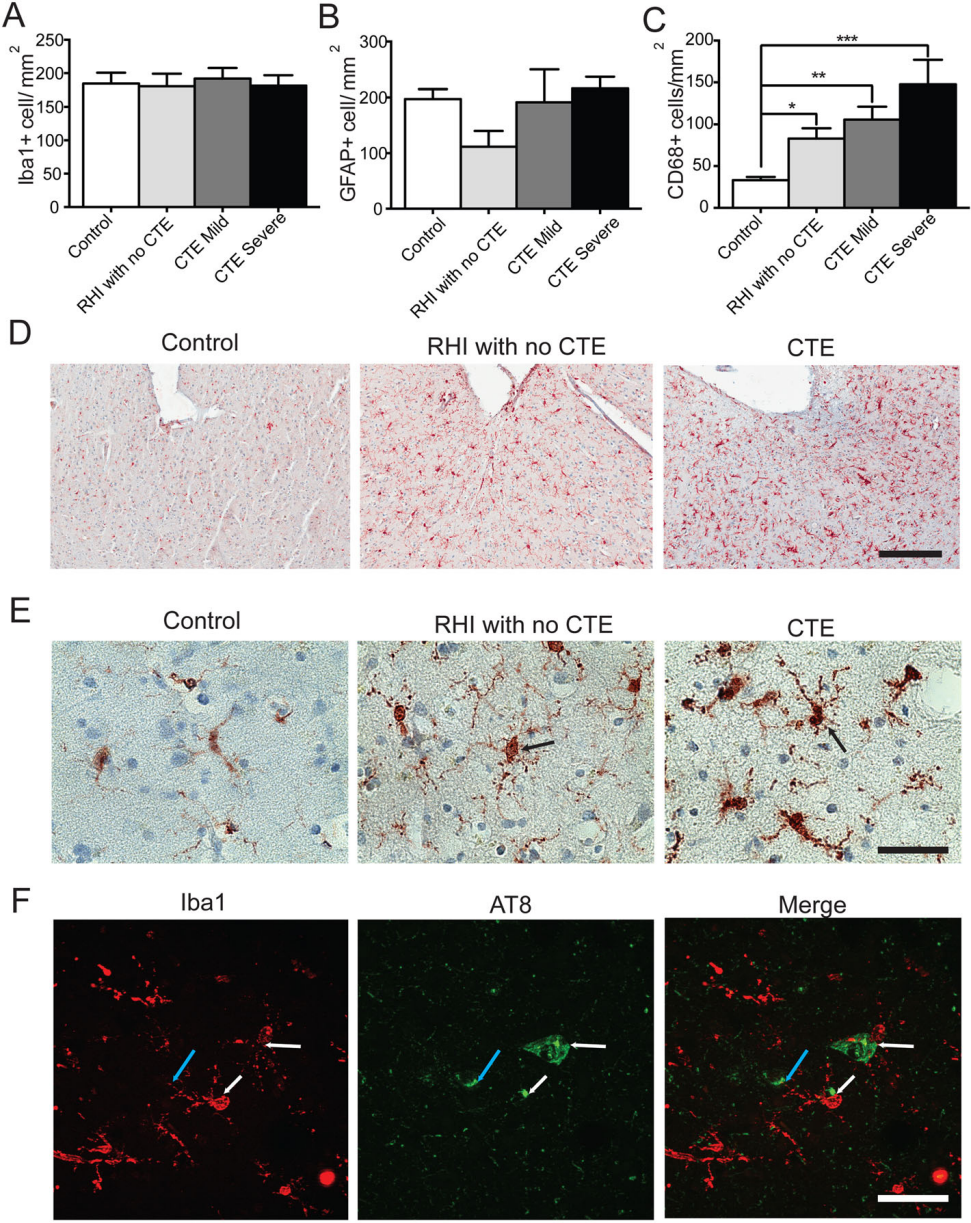
Glial morphology, but not overall cell number, changes during CTE pathological stage progression. **a**-**c** Quantification of the total number of Iba1 positive (**a**), GFAP positive (**b**), and CD68 positive cells present at the depths of the sulci in the dorsolateral frontal cortex. **d** Representative images at 10× (**d**) and 63× (**e**) magnification of Iba1 depicting the morphologic change of microglia into activated, reactive morphology (arrows). **f** Representative image of Iba1 immunoreacive microglia surrounding clusters of AT8 immunoreactive cells. White arrows denote Iba1 cell body near AT8 aggregates. Blue arrow denotes microglia process contacting AT8 immunoreactive neuron. Data displayed as mean ± SEM, scale bars represent 300 μm (**d**) and 50 μm (**e**,**f**), *p ≤ 0.05, **p ≤ 0.01

Overall, there was no statistical difference in the density of total Iba1 immunoreactive microglia (Fig. 1a) or GFAP immunoreactive astrocytes (Fig. 1b) between groups. However, the cellular density of CD68 immunoreactivity, a lysosomal marker that labels phagocytic/activated microglia [19], was significantly increased in subjects with a history of RHI, with or without CTE, when compared to controls (Fig. 1c). Consistent with increased density of CD68 immunoreactive cells, microglia also exhibited a phenotypic change (Fig. 1d). High power imaging revealed a larger cell body and more numerous, shorter processes, indicative of a reactive phenotype in RHI subjects (Fig. 1e, arrows). Iba1 positive activated microglia were frequently found surrounding AT8 immunoreactive neurons (Fig. 1f, white arrow) with extended microglial processes that contacted the AT8 positive neuronal soma (Fig. 1f, blue arrow).

### The density of CD68 positive cells predicts tau pathology

**Fig. 2:**
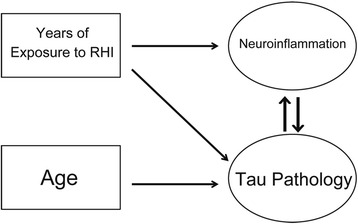
Pathway analysis of CTE associated variables. Visual representation of significant interactions from the simultaneous equation regression analysis. Rectangles represent predictor variables while circles represent outcome variables. Arrows denote a significant direct effect

To test the hypothesis that RHI exposure and age have effects on microglial activation and ptau pathology and to test for both direct and indirect effects, we performed a simultaneous equations regression model analysis. This form of structural equation modeling is useful to account for potential feedback loops. Additionally, the cumulative effect through a specific pathway of multiple variables, as well as individual direct effects can be modeled and tested. The stability coefficient of the model was 0.09, which is less than one and means that the condition for converged total and indirect effects was satisfied. The model fit was excellent with the Goodness of Fit Index (GFI) equal to 0.99. Years of RHI was used as the exposure metric instead of the number of concussions due to linear regression modeling demonstrating CD68 having a significant relationship with RHI (β = 0.418, *p* < 0.001), but not number of concussions (β = 0.128, *p* = 0.266). This is consistent with previously published data [10]. This model tested the direct and total effects of predictor variables (age and years of exposure to RHI) on select outcomes (CD68 cell density and AT8 tau density). The statistical outcomes of this model are provided in Table 2 and a visual representation of the data is provided in Fig. 2. Overall, the number of years of exposure to RHI had a significant direct effect on both AT8 density (β = 0.4614, *p* < 0.0001) and CD68 cell density (β = 0.3911, *p* < 0.0001). Comparing the standardized effects demonstrated that the effect of RHI exposure on AT8 tau density was 86 % direct and 14 % via increased CD68 immunopositive cell density (Table 2). Age also demonstrated a significant positive effect on AT8 density (β = 0.5125, *p* < 0.0001) but not CD68 cell density (β = −0.0195, *p* = 0.84). In addition, CD68 cell density had a significant direct positive effect on AT8 tau pathology (β = 0.1759, *p* < 0.0001). AT8 density also had a direct positive effect on CD68 cell density (β = 0.0498, *p* < 0.0001), suggesting a positive feedback loop. The magnitude of the standardized effect of CD68 cell density on AT8 density was 3.5 times greater than the effect of AT8 tau pathology on CD68 cell density. Overall, duration of RHI had an effect on severity of AT8 tau pathology that was both direct and via increased CD68 cell density. Furthermore, the observed increase in CD68 cell density was independent of age and directly associated with duration of exposure to RHI and severity of AT8 immunopositivity (Table 2).

**Table 2:** Simultaneous equation regression model of the direct and total effects of age and years of exposure to RHI on CD68 cell density and tau pathology (AT8 tau density)

	Standardized direct effects		Standardized total effects	
	Effect/Std error/*t* value/*p* value		Effect/Std error/*t* value/*p* value	
	CD68 Density	AT8 Tau Density	CD68 Density	AT8 Tau Density
Age	−0.0195	0.5125	0.006044	0.5136
	0.1003	0.0682	0.1009	0.0691
	−0.1948	7.5184	0.0599	7.4306
	0.8456	**<.0001**	0.9523	**<.0001**
RHI Exposure (Years)	0.3911	0.4614	0.4178	0.5349
	0.0852	0.071	0.0917	0.0681
	4.5911	6.4999	4.5551	7.8586
	**<.0001**	**<.0001**	**<.0001**	**<.0001**
CD68 Density		0.1759		0.1774
		0.0499		0.0507
		3.5271		3.5005
		**<.0001**		**<.0001**
AT8 Tau Density	0.0498		0.0503	
	0.0112		0.0114	
	4.4363		4.4058	
	**<.0001**		**<.0001**	

Simultaneous equation regression was used to analyze the direct and total effects. Bolded numbers represent significant *p* values. Visual pathway analysis can be found in Fig. 2

### Neuroinflammation indirectly correlates with dementia

**Fig. 3:**
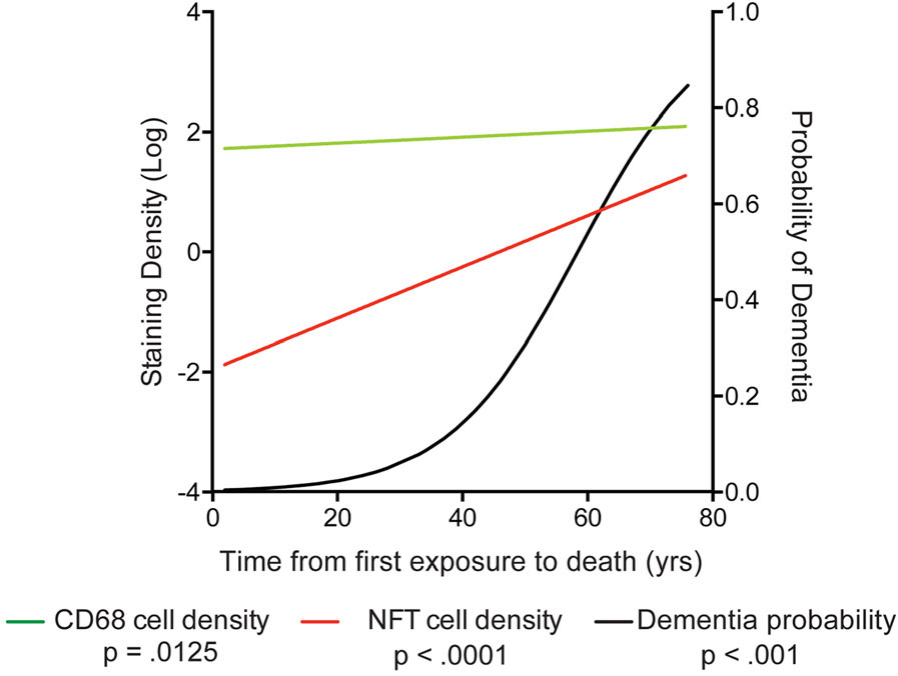
A model of the progression of neuroinflammation, ptau pathology, and probability of developing dementia in CTE. The regression lines for CD68 cell density, NFT density, and the probability of developing dementia were plotted within a single graph and correlated with time from first exposure to death (years). Binary logistic regression beta values were used to generate the probability curve for dementia

To compare the time course of neuroinflammation, NFT aggregation, dementia, and to assess possible correlations, variables were graphed together based on the time from first exposure to death (Fig. 3). The presence of dementia was based on medical record review or next of kin interview denoting a diagnosis of dementia made by a physician during life. Binary logistic regression demonstrated that longer time from first exposure to death was significantly associated with an increased risk of developing dementia (OR = 1.102, *p* < 0.001). A probability curve was generated based on the logistic regression and is plotted in Fig. 3 (black line). In addition, time from first exposure to death significantly predicted CD68 density (β = 0.005, *p* = 0.0213) (green line) and separately, neurofibrillary tangle (NFT) density (β = 0.043, *p* < 0.0001) (red line) (Fig. 3). The slopes (CD68: 0.005020 ± 0.002125, NFT: 0.04274 ± 0.005880) suggest that there is a 0.5 % increase of CD68 positive cells/mm^2^ per year and a 4.2 % increase in NFTs/mm^2^ per year in CTE.

To test if neuroinflammation was a predictor of dementia, a binary logistic regression demonstrated dementia was significantly predicted by CD68 (OR = 1.010, *p* = 0.011) independent of age (OR = 1.055, *p* = 0.007). Interestingly, when ptau pathology was included into the model, NFT density (OR = 1.12, *p* = 0.007) significantly predicted dementia independent of age (OR = 1.050, *p* =0.039), but CD68 density (OR = 1.007, *p* = 0.079) was no longer a significant predictor. This suggests that the effect CD68 has on dementia is mediated through the development of tau pathology.

## Discussion

We show that increased neuroinflammation, as evidenced by increased CD68 cell density and enhanced microglia reactive morphology, was associated with more severe AT8 immunopositive ptau pathology in the DLF cortex of subjects neuropathologically diagnosed with CTE. Furthermore, duration of RHI exposure, as defined by the years of football played, predicted greater density of CD68 positive inflammatory microglia in American football players with and without CTE pathology. A simultaneous equations regression model demonstrated that exposure to RHI had a significant effect on ptau pathology that was both direct and indirect (via increased CD68 cell density). Last, we found that increased neuroinflammation was related to the risk of a subject being diagnosed with dementia and that the relationship was mediated through ptau, independent of age. Altogether, this suggests that increased neuroinflammation as a consequence of prolonged RHI exposure may play an important role in the development of ptau pathology in CTE. To our knowledge this is the first data directly linking neuroinflammation to the development of ptau pathology in CTE.

Although moderate/severe TBI has been observed to result in long lived neuroinflammation [20], even mild head impacts may lead to brain injury, including multifocal traumatic axonal disruption, that is concentrated in regions such as the white matter-gray matter junction, around blood vessels, and at the depth of the cortical sulci [12, 21–23]. Multifocal traumatic axonal injury may be an early trigger for neuroinflammation [24], with microglial activation and initiation of an immune response that serves to repair or limit the damage [25]. The interval between injuries also impacts the long-term CNS response [26]. After isolated or infrequent mild TBI the induced neuroinflammatory response may dissipate as the CNS tissue is repaired. However, repetitive injuries, particularly RHI that occur during a short interval that do not allow for complete recovery, may lead to a persistent proinflammatory state. Furthermore, neuronal death resulting from mechanical injuries has the potential to result in cell lysis and exposure of intracellular components into the extracellular environment. Intracellular proteins like HMGB1, ATP, and other damage associated molecular patterns (DAMPS) can trigger a rapid innate immune response and induce chronic neuroinflammation [27].

Numerous studies have demonstrated that neuroinflammation occurs acutely following TBI [11, 28–30]. In addition, mouse models that mimic the repetitive injury observed in humans have shown that glial changes and neuroinflammation precede ptau pathology [31, 32]. We also observed elevated CD68 positive cell density in RHI-exposed subjects in the absence of any ptau pathology (Fig. 1c). These individuals were younger and the duration of exposure to RHI was less than the CTE group; thus, they may represent individuals in a prodromal state.

The simultaneous equations regression analysis is a form of structural equation modeling that incorporates feedback loops [33] and is therefore well suited to model the effects of interacting pathologies. In a cohort of subjects with and without exposure to RHI, we found that RHI exposure had a cumulative effect on the extent of ptau pathology that was both direct and mediated through increased CD68 cell density (Table 2, Fig. 2). An activated microglial-mediated increase in ptau is consistent with previous work demonstrating that neuroinflammation increased the activity of GSK3β and p38MAPK, both of which are involved in tau hyperphosphorylation [14] and promote ptau pathology [34]. The simultaneous equations regression model also demonstrated that both CD68 and ptau had a significant effect on each other (Fig. 2). This is in agreement with previous studies in mouse models that have shown an inflammatory mediated increase in ptau as well as tau induced neuroinflammation [35]. Our data suggest that a similar feedback mechanism may be present in human CTE. Thus, down regulation of chronic microglia activation might represent an important therapeutic target for CTE. Furthermore, there was a significant direct effect of RHI exposure on increased ptau pathology independent of CD68. The mechanisms underlying this increase in ptau following RHI are unknown, but might involve calcium influx and activation of kinases which are known to occur after mild traumatic brain injury [26]. In addition to its association with RHI exposure and ptau pathology, CD68 cell density in the DLF appears to plays an indirect role in the development of dementia in CTE, mediated through ptau pathology. A binary logistic regression demonstrated dementia was significantly predicted by CD68 independent of age, but that association was eliminated when a measure of ptau pathology was included in the model. Our data suggest that increased activated microglia occur early following RHI, followed by a later, but steeper increase in ptau pathology, and finally cumulating in dementia in some cases (Fig. 3). In support of this, cumulative RHI exposure has recently been associated with cognitive impairment later in life [8] and TSPO PET ligands for activated microglia have shown elevated activity in retired NFL players at risk for CTE [36]. Activated microglia and persistent neuroinflammation are one mechanism by which RHI exposure may drive the development and spread of ptau pathology and lead to dementia in CTE.

There are several limitations to our study. There is selection bias in an autopsy-based study of individuals whose brains are donated by the family, and the subjects may not represent the population as a whole. Additionally, clinical and RHI exposure histories are obtained retrospectively and are subject to bias. Future longitudinal, prospective studies will be needed to verify these results. For the simultaneous equations regression analysis, CD68 and AT8 densities were treated as both outcomes and predictors to avoid the “endogeneity” problem (i.e., (1) a linear regression model that has independent variables as predictors would give invalid inference due to the significant correlation between the predictor and the error term, and (2) their coefficients would be biased) [37]. To address the inference and bias problems caused by endogeneity, we used simultaneous equation models, which are widely used in econometrics [33]. For cell quantitation we used the Aperio nuclear algorithm to determine the number of stained cells. In contrast to design based stereological methods, the nuclear algorithm lacks 3-dimensional volume measurements and does not provide an estimate of the total number of microglia [18]. However, we obtained similar results when using the positive pixel algorithm. Finally, while CD68 is typically associated with a more inflammatory, phagocytic microglia phenotype [38], glial activation is too complex and dynamic to be completely recapitulated by one marker. Future studies will be needed to explore additional markers and inflammatory cytokines to better define the neuroinflammatory phenotype in CTE.

## Conclusions

Here, we showed for the first time that neuroinflammation was associated with greater ptau pathology in CTE. Furthermore, we demonstrated that the duration of RHI exposure predicted increased activated microglial cell density that, in turn, was directly associated with ptau pathology (Fig. 2). Lastly, our data suggest that neuroinflammation might partially underlie clinical symptoms of CTE, like dementia, through their effect on ptau pathology. Overall, the potential central role of increased neuroinflammation in CTE development and progression suggests that inflammatory molecules may be important diagnostic or predictive biomarkers as well as promising therapeutic targets in CTE.
